# Toward Optimized Proteomics Analysis to Detect Bacterial and Host Proteins in Calcified Coronary Artery Plaques

**DOI:** 10.1021/acs.jproteome.6c00422

**Published:** 2026-09-14

**Authors:** Kirsikka Musta, Markus J. T. Ojanen, Maria Stensland, Jonna Kankaanranta, Terho Lehtimäki, Pekka J. Karhunen, Tuula A. Nyman, Vesa P. Hytönen

**Affiliations:** † Faculty of Medicine and Health Technology, 7840 Tampere University, Tampere 33520, Finland; ‡ Department of Immunology, University of Oslo and Oslo University Hospital, Oslo 0372, Norway; § Fimlab Laboratories, Tampere 33520, Finland; ∥ Department of Clinical Chemistry, Fimlab laboratories Tampere 33520, Finland; ⊥ Finnish Cardiovascular Research Center - Tampere, Faculty of Medicine and Health Technology, Tampere University, Tampere 33520, Finland

**Keywords:** Bacteria, Coronary Artery Disease, Atherosclerosis, Mass Spectrometry, Calcification, Metaproteomics

## Abstract

Coronary calcification is the final stage in coronary heart disease, associated with significantly increased morbidity and mortality. The molecular pathology of calcification and especially events triggering calcification are poorly understood. Metagenomic and molecular microbial techniques have identified the presence of oral biofilm-producing and dental calculus-forming bacteria in atherosclerotic plaques, suggesting that they may be involved in calcification. To enable studying this at the proteome level, we compared four different protein extraction methods to find a robust and reliable method for the identification and quantification of human and bacterial proteins from 10 calcified arterial plaques. We identified 3 913 human proteins and 24 bacterial proteins. These findings provide further support for the presence of bacterial proteins in calcified areas of coronary artery plaques, with Fusobacteria being the most abundant bacterial protein source. Comparisons of the extraction methods showed that the urea-based extraction method was most optimal due to its simplicity and reproducible identification of a high number of proteins, which makes it suitable for further large-scale analysis of coronary artery plaque samples.

## Graphical abstract

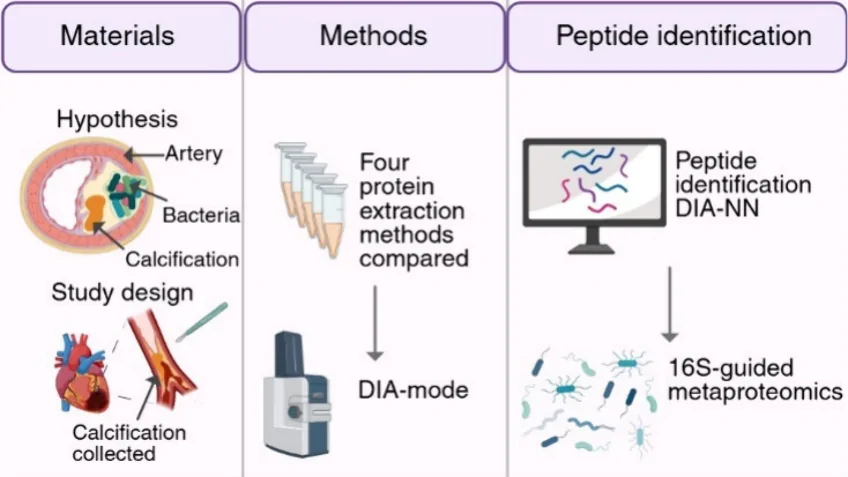

## Introduction

Coronary artery disease is the leading cause of death in the world.^1^ Coronary artery calcification (CAC)—the end stage of coronary artery disease—was previously thought to be a passive phenomenon.^2^ The current view is that it is a complicated biological process characterized by vascular smooth muscle cells (VSMCs) trans-differentiating into osteogenic phenotype. These cells then release matrix vesicles (MVs) which serve as nucleation sites for the development of hydroxyapatite (HA) crystals that become deposited in the gaps of the collagen fibrils thus forming the calcified coronary plaque that resembles bone in its chemical composition.^3,4^ Dying SMCs and macrophages also participate in the process by releasing apoptotic bodies which serve as a nucleation site for HA.^5–7^ The exact molecular mechanisms activated during the onset of coronary calcification are not fully understood.^5^ Even less is known about the events that trigger the onset of calcification.^6^

There is increasing evidence showing that inflammation is involved in the early stages of coronary artery calcification.^8^ The present view on the pathogenesis of atherosclerosis entails that it is a chronic subclinical inflammation driven by oxidized low-density lipoprotein (oxLDL).^9,10^ Previous studies, including our own work, have used molecular microbiological approaches such as metagenomics and immunohistochemistry to identify bacterial DNA and antigens originating from oral bacteria in atherosclerotic plaques.^11–14^ Combined, these results suggest that oral bacteria may have a role in chronic inflammation linked to atherosclerosis. Oral bacteria are known to build up a biofilm on the surface of the teeth.^15^ This biofilm can calcify and form dental calculus.^16^ We have recently found that oral viridians group streptococci participate in the formation of a biofilm inside coronary plaque and that innate immune system does not detect this biofilm.^11^ However, it has not been studied whether the bacterial biofilm could be involved in the calcification of coronary atherosclerosis. If that is the case, bacterial proteins or their remnant peptides should be identified in the calcified coronary plaque along with host (human) proteins. A better understanding of molecular components of calcified plaques could open possibilities for the development of novel therapeutic approaches.

Mass spectrometry (MS)-based proteomics is a widely utilized, robust, and sensitive method to identify and quantify proteins in different clinical samples. It enables identification of proteins from diverse biological origins, including host and microbial species,^17^ and is therefore ideal for analyzing the role of previously unknown factors in calcification. Protein identification by LC-MS/MS can be done using either data-dependent or data-independent acquisition (DDA or DIA). In DDA, only the most abundant ions are fractionated in the second stage of MS. This enables easier identification of spectra but poses challenges when working with low-abundance proteins. In contrast, in DIA, all the ions are fractionated in the second stage of the MS, producing highly convoluted spectra. While this previously posed difficulties in data analysis of the MS/MS spectra, modern-day software such as DIA-NN^18^ and Spectronaut have been developed to address this issue.

In recent years, metaproteomics has emerged as a new field of study. Similar to metagenomics, where the genomes of microbes are being studied, metaproteomics studies the proteomes of microbes in multispecies samples. Traditional microbial proteotyping relies on the analysis of microbial proteins for species identification and includes MALDI-TOF-based methods as well as more sensitive LC-MS/MS-derived approaches used in clinical settings.^19–22^ In contrast, metaproteomics seeks to characterize the functional activity of microbial communities through comprehensive analysis of the proteomes of the microorganisms present in a sample.^23^ Recent developments increasingly blur this separation, as proteotyping workflows now detect antimicrobial resistance and virulence factors,^20^ while metaproteomic pipelines commonly include taxonomic inference.^24,25^ Accordingly, metaproteomic workflows are typically optimized for broad and deep proteome characterization across complex microbial communities. However, the majority of the metaproteomics studies so far have been conducted using DDA.^24–29^ DIA has also been utilized in some recent metaproteomic studies conducted using cultured bacteria,^30–32^ fecal samples,^33–35^ or other sample material such as saliva^36^ where bacteria are present in high amounts.

Very few proteomic studies have been done on calcified coronary arteries. To our knowledge, only Herrington et al.^37^ have used MS-based proteomics to characterize calcified coronary samples focusing only on human proteins in early atherosclerosis and observed differences in mitochondrial pathways. While calcified samples such as dental calculus have been studied using DDA,^38–41^ to the best of our knowledge, there are no previous DIA-based MS studies for calcified samples. We chose to use DIA to improve the identification of bacterial proteins, particularly as they are expected to be present at low abundances. Here, we have compared four different protein extraction methods to establish a robust and reliable protocol for the identification of human and low-abundance bacterial proteins from calcified arterial samples, enabling high-throughput proteome analysis of coronary samples.

## Materials and Methods

### Coronary Plaque Samples

Ten calcified right coronary artery (RCA) samples from different subjects were chosen from Tampere Sudden Death Study (TSDS) series. Samples were collected during medicolegal autopsies between years 2010 and 2015 (ethical permission number R09097).^11,42^ The age range was 64–88 years (mean 77.2 years), the stenosis percentage of the right coronary arteries (RCA) ranged from 47.8% to 100% (mean 79.0%) The samples were collected 3–11 days post-mortem (mean 5.5 days) and stored at −80 °C prior protein extraction.

### Mechanical Homogenization

The calcified area of the coronary plaques was carefully separated from the surrounding soft tissue with a scalpel. The calcifications were then cut to smaller approximately 2 mm × 2 mm pieces before homogenization using a drill (Dremel, cat. no. 8220-1/5, Mexico). To ensure consistency across methods, each sample was subdivided into four equal fractions of approximately 100 mg for the various protein extraction methods.

### Protein Extraction

The samples were treated according to four previously published protein extraction methods^37,40,41,43^ with minor modifications. Brief descriptions of each method are given below, and a full list of used reagents is provided in the .

In **method 1** ^40^ the sample was first washed with 0.5 M EDTA for 5 min. Then the sample was decalcified with 0.5 M EDTA for 7 days in constant 20 rpm rotation at RT. Based on our preliminary experiments the sample volume was reduced to 250 μL compared to original publication to achieve higher protein concentration. After decalcification, majority of the EDTA solution was removed and 50 μL of EDTA was left with the calcification sample. 45 μL of B-PER (Bacterial Protein Extraction Reagent) and 5 μL 20% SDS were added to the sample. The sample was then shaken for 15 min at RT, and any remaining solid material was removed with a disposable laboratory spatula. Gel Aided Sample Preparation (GASP)- steps used in^40^ were omitted. The sample was stored at −20 °C.

In **method 2** ^41^ the sample was washed with 0.5 M EDTA and then treated with 0.5 M EDTA at 4 °C for 24 h. The supernatant was moved to a clean sample tube; the sample was named 2a and stored in −20 °C. The remaining calcification was incubated in 6 M guanidine hydrochloride (GdnHCl), 100 mM Tris at pH 7.4 at 4 °C for 18 h. The solid material was removed with a disposable laboratory spatula, and the sample was named 2b and stored at −20 °C.

In **method 3** ^37^ the sample was placed in solution containing freshly prepared 8 M urea, 2 M thiourea, 4% CHAPs and 1% dithiothreitol (DTT) for 15 min at RT using shaking. The solid material was removed with a disposable laboratory spatula, and the sample was stored at −20 °C.

In **method 4** ^43^ two homogenization steps were originally included, but the first homogenization step with metal beads was omitted due to unified homogenization protocol used, described above. The second homogenization step using zirconium beads in 5% sodium deoxycholate was performed with Bullet blender storm 24. The sample was then sonicated for 30 min with 5 min on/off cycle in the highest setting. The sample was centrifuged for 10 min in 15 115 g. The supernatant was moved to a clean sample tube, and the sample was stored at −20 °C.

### PAC Purification and Protein Digestion

All samples underwent protein aggregation capture (PAC) purification using MagReSyn AmineBeads according to manufacturer’s protocol.

Briefly, the proteins extracted from tissue homogenates were precipitated with 70% acetonitrile onto magnetic beads. The beads were washed with 100% acetonitrile, 70% ethanol and then resuspended in 100 μL of 50 mM ammonium bicarbonate containing 10 mM DTT for 37 °C for 30 min. A small sample was taken for bicinchoninic acid assay (BCA) and SDS-PAGE analyses. Then, to alkylate proteins, 2.7 μL of 550 mM iodoacetamide (IAA) was added and the sample was incubated at RT in the dark for 30 min. 0.5 μg trypsin was added to each sample for overnight on-beads protein digestion at 37 °C. Finally, the sample was acidified with 0.1% (v/v) of formic acid to stop the digestion.

### Determination of Protein Concentration and SDS-PAGE Analysis

The protein concentration was measured using reducing agent compatible Pierce Microplate BCA Protein Assay Kit and Spark Multimode Microplate Reader (Tecan, Switzerland). One sample from each method was chosen for SDS-PAGE analysis. 1.8–5 μg of protein from each sample was taken to ensure that the samples are within the dynamic range of silver staining. Silver staining was performed using Pierce Silver Staining kit.

### LC-MS/MS Analysis

The resulting peptides after trypsin digestion were desalted using C18-tips according to manufacturer’s instructions. Purified samples were dried using MiVac concentrator (Genevac). Peptides were dissolved in 0.1% formic acid and analyzed by a nanoElute UHPLC coupled to a timsTOF PRO2 mass spectrometer (Bruker Daltonics, Bremen, Germany) via a CaptiveSpray ionsource. Peptides were separated on a 25 cm reversed-phase C18 column (1.6 μm bead size, 120Å pore size, 75 μm inner diameter, Ion Optics) with a flow rate of 0.3 μL/min and a solvent gradient from 0 to 35% B in 60 min. Solvent B was 100% acetonitrile in 0.1% formic acid and solvent A 0.1% formic acid in water. The timsTOF PRO2 mass spectrometer was operated in DIA-PASEF mode. Mass spectra for MS were recorded between *m*/*z* 100 and 1700. Ion mobility resolution was set to 0.85–1.30 V·s/cm over a ramp time of 100 ms. The MS/MS mass range was limited to *m*/*z* 475–1000 and ion mobility resolution to 0.85–1.30 V s/cm to exclude singly changed ions. The estimated cycle time was 0.95 s with 8 cycles using DIA windows of 25 Da. Collisional energy was ramped from 20 eV at 0.60 V s/cm to 59 eV at 1.60 V s/cm.

One sample prepared with method 1 was excluded from the database search due to low MS signal intensities. Raw data files from LC-MS/MS analyses were submitted to DIA-NN (version 1.9.2) for protein identification and label-free quantification using *in silico*-generated spectral libraries from human and selected bacterial FASTA files. All the FASTA-files (human and bacterial) were retrieved from UniProt by downloading complete set of protein sequences for each taxon. To ensure computational feasibility, only a defined set of bacterial species were included from some bacterial genera (Table 1), rather than complete genus-level databases. This selection was guided by genus-level 16S sequencing data^14^ which identified bacterial taxa present in coronary artery samples in our cohort, and further refined based on literature.^15,44–46^ To control for the rate of false discoveries, several control bacteria not present in the 16S-sequencing results^14^ were added to the database. The control bacteria included environmental bacteria (*Azospirillum brasilense*, *Buchnera aphidicola*, *Serratia symbiotica*, *Streptomyces coelicolor*, and *Synechocystis sp. PCC 6803*) as well as other commensal or pathogenic human bacteria that to our knowledge have not been detected in coronary atheromas (*E. coli O157:H7 strain*, *Pseudomonas aeruginosa*, *Gardnerella vaginalis*, *Staphylococcus aureus*, and *Roseburia intestinalis*). Full list of target species is provided in Table 1 and full FASTA databases used in this study are available from the PRIDE-accession (PXD078118). The total size of the bacterial databases was 854 817 sequences, 531 687 sequences belonging to the 16S-sequencing based bacteria and 323 130 sequences belonging to the control bacteria.

**Table 1:** Bacterial Species Used in Database Searches^a^

Target species		
Genus	Selected species (# of sequences)	Control species (# of sequences)
*Fusobacterium*	The entire genera (170 227)	*Azospirillum brasilense* (42 898)
*Veillonella*	The entire genera (72 078)	*Buchnera aphidicola* (30 311)
*Prevotella*	intermedia, melaninogenica (35 996)	*Serratia symbiotica* (8 538)
*Streptococcus*	mutans, gordonii, mitis, sanguinis (191 514)	*Streptomyces coelicolor* (8 683)
*Lactobacillus*	gasseri, rhamnosus (32 521)	*Synechocystis sp. PCC 6803* (3 510)
*Lacticaseibacillus*	casei (9 597)	*E. coli O157:H7 strain* (18 196)
*Limosilactobacillus*	fementum (17 032)	*Pseudomonas aeruginosa* (72 582)
*Clostridium*	perfringens (2 722)	*Gardnerella vaginalis* (21 453)
		*Staphylococcus aureus* (88 773)
		*Roseburia intestinalis* (28 186)

a Target species based on 16S-sequencing results performed for arterial samples from same clinical sample collection^14^.

Carbamidomethyl (C) was set as a fixed modification. Trypsin without proline restriction cleavage option was used, with one allowed miscleavage, and peptide length range was set to 7–30 amino acids. The mass accuracy was set to 15 ppm, and the precursor false discovery rate (FDR) 0.01 (1%) was allowed. Raw proteomics data and FASTA libraries used in in silico spectral library generation are available in the PRIDE repository with project accession PXD078118.

### Filtering

BLASTp run against the human proteome (nr-database) was performed for the bacterial peptides. Isoleucine and leucine residues were replaced with the ambiguous amino acid code J, because they are indistinguishable by mass spectrometry due to their identical masses. Peptides that were 100% matches to human were filtered from downstream analyses. Hits belonging to control bacteria were filtered from the downstream analyses.

### Bioinformatics

Proteomic data were analyzed in R 4.5.2,^47^ the *Peptides*,^48^ *GO.db*,^49^ and *PCAtools* ^50^ packages. The GO terms for the identified bacterial proteins were fetched from OrthoDB. The fully reproducible code is available in the .

## Results and Discussion

Calcification of the coronary arteries is the final stage of coronary artery disease. While previous proteomics studies, such as Herrington and colleagues,^37^ have identified human proteins from coronary artery samples, to our knowledge, the calcified arteries have only been analyzed by Nehme and colleagues,^43^ and their research focused on carotid arteries. To enable reproducible identification of human and bacterial proteins from calcified plaques, we compared four different protein extraction methods^37,40,41,43^ for protein isolation in parallel from 10 clinical samples and performed LC-MS/MS analysis with the DIA-PASED method for all of them. The workflow is depicted in Figure 1.

**Figure 1:**
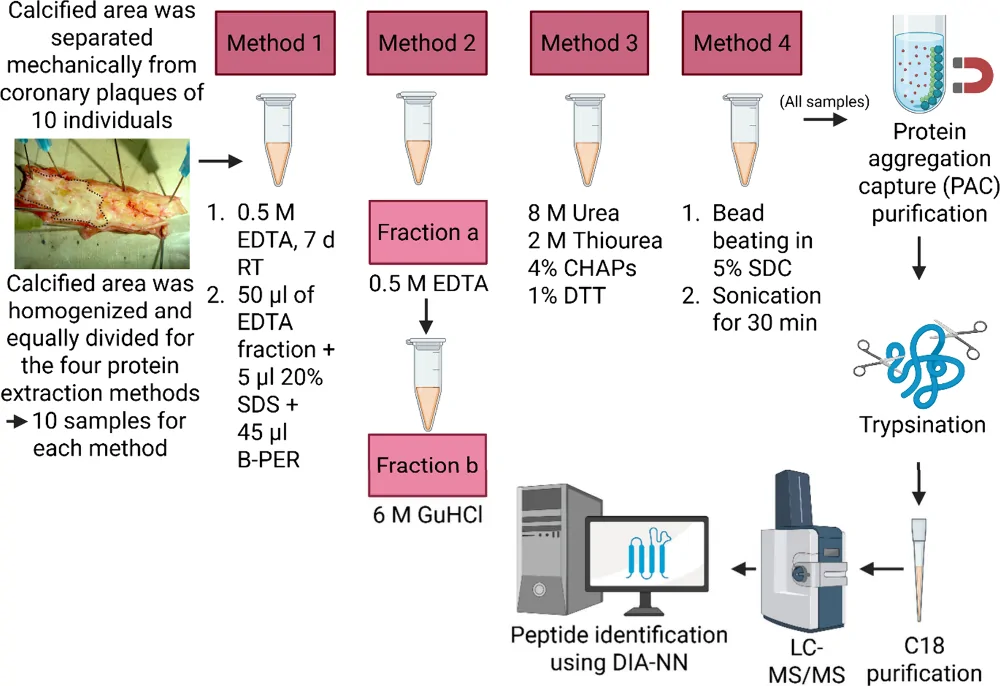
Workflow for the protein extraction and MS-based identification of proteins. Created with BioRender.com.

### Protein Yields

Silver stained SDS-PAGE (Figure 2A) revealed several proteins in each sample from each method. The yields for the method 1 ranged from 96 μg/mL to 938 μg/mL (mean 389 μg/mL, standard deviation 267 μg/mL), method 2a from 66 μg/mL to 1 159 μg/mL (mean 380 μg/mL, standard deviation 361 μg/mL), method 2b from 97 μg/mL to 680 μg/mL (mean 410 μg/mL, standard deviation 193 μg/mL), method 3 from 563 μg/mL to 27 724 μg/mL (mean 8 753 μg/mL, standard deviation 894 μg/mL) and for the method 4 from 189 μg/mL to 3 348 μg/mL (mean 1 386 μg/mL, standard deviation 992 μg/mL). The variability was likely caused by the heterogenicity inherent to biological material. Due to low protein yield, protein quantification failed in one sample in method 1, three samples in method 2a and 2b, whereas all samples analyzed with method 3 and 4 were successfully quantified.

**Figure 2:**
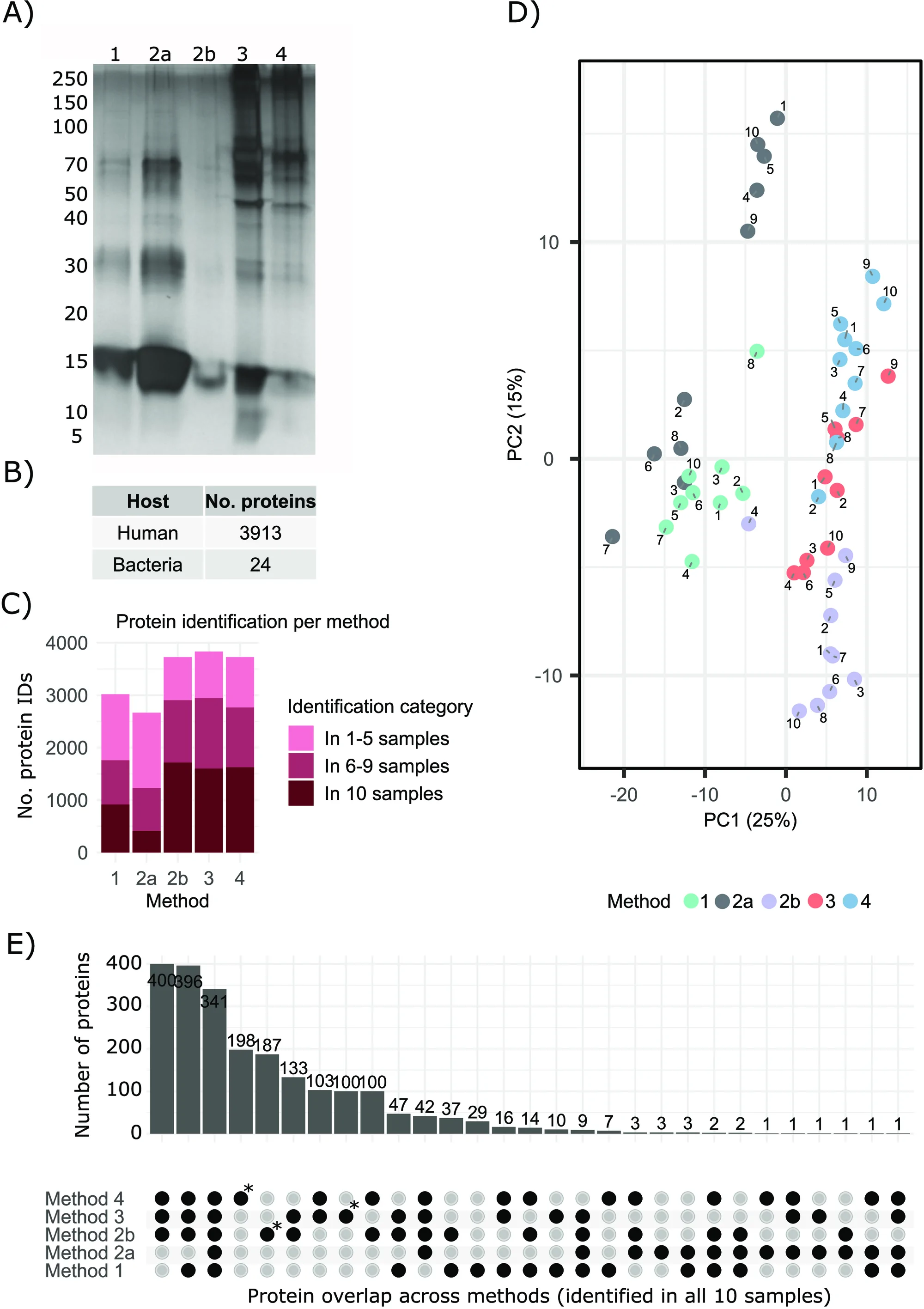
Overview of the proteomics results from the different protein extraction methods A) Silver-stained SDS-PAGE. 3.5 μg, 5.3 μg, 1.8 μg, 5.1 μg, and 4.6 μg of protein extract was loaded to the gel, respectively. B) Total number of identified human and bacterial proteins. C) Number of identified proteins from different protein extraction methods showing all identified proteins and reproducible identification (1–5 samples, 6–9 samples and 10/10 samples (1–5, 6–8, and 9 samples in method 1 where one sample was excluded due to low intensity signals in MS analysis). D) Principal component analysis of proteomic data. Subjects are numbered. E) UpSet plot showing overlap of protein identification between methods for human proteins identified in all 10 samples. * = protein identified uniquely in each method analyzed more in .

### Identified Proteins

In total, 3 913 human proteins and after filtering 24 bacterial proteins were identified (Figure 2B). There was clear variation between the number of identified proteins across the different protein extraction methods (Figure 2C). Method 3 yielded the highest number of identifications, in total 3 826 proteins, whereas method 2a resulted in the lowest number, with 2 662 proteins identified. The number of proteins identified in all parallel samples was similar in methods 2b, 3, and 4. In comparison, Nehme and colleagues^43^ identified 621 proteins from 32 paired samples, and Herrington and colleagues^37^ identified 1 925 proteins from 200 arteries, which was less than what we identified. Previous studies, however, used data-dependent acquisition with older generation MS instruments instead of the DIA-PASEF workflow utilized here.

Principal component analysis (Figure 2D) shows that replicate samples from each protein extraction method form distinct groups, and no grouping based on different subjects is seen. Furthermore, EDTA-based methods 1 and 2a group apart from other methods. The UpSet plot (Figure 2E) of proteins identified in all 10 parallel samples shows that this is likely due to the fact that the methods 2b, 3, and 4 identified largely the same proteins whereas the EDTA based methods (1 and 2a) had significantly lower number of shared identifications with the other methods. In method 2 there were two fractions: EDTA-fraction 2a which aimed to extract soluble proteins and GuHCl-fraction 2b which aimed to extract the leftover nonsoluble proteins. Based on the UpSet plot of all identified proteins () this was largely not the case, as the proteins identified with methods 2a and 2b were largely overlapping.

The human proteins that were identified in all 10 samples by only one extraction method (2b, 3, or 4) were studied more closely to evaluate the effects of different chemical bases on the extraction methods. There was no difference in the proteins between the methods based on membrane association, but there were statistically significant differences in molecular weight, isoelectric point, and aliphatic index (). Method 3 identified proteins with a lower isoelectric point, while method 4 identified proteins with a higher aliphatic index.

### Bacterial Proteins Were Identified in Coronary Artery Plaques

To study the presence of bacterial proteins in coronary arteries, we included selected bacterial proteomes in the database search based on our previous 16S sequencing results^14^ as well as selected control bacteria to control for false positive identifications. In the DIA-NN search, 63 different bacterial proteins were identified, but in BLASTp run against human proteome, 25 of these identified peptides were 100% matches to human protein sequences (Figure 3A,B). Most of these belonged to *Buchnera aphidicola*, a maternally transmitted obligate insect endosymbiont incapable of surviving outside of its host.^51^ Therefore, those peptides, belonging to proteins such as DnaK, likely correspond to false positives. Additionally, 14 were proteins that belonged to other control bacteria. For details, see the Supporting Information ZIP. Both categories were omitted from downstream analyses, resulting in 24 bacterial proteins, and 13–20 bacterial proteins were identified by each method (Figure 3C). All of the bacterial proteins in our data set were identified with one peptide.

**Figure 3:**
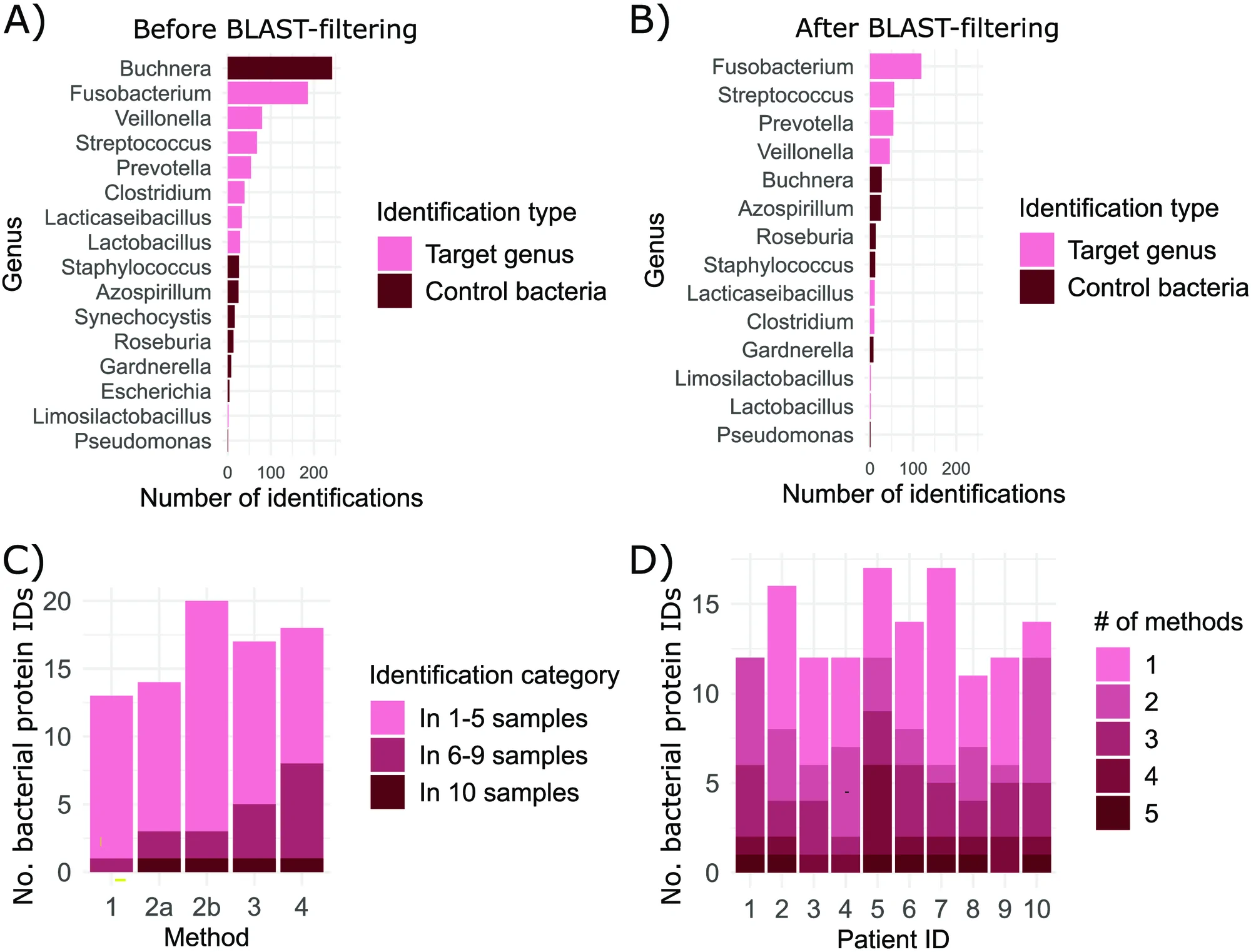
Overview of identified bacterial proteins across methods. A) Number of bacterial proteins multiplied by the number of detections in each genus before BLAST-filtering. B) Number of bacterial proteins multiplied by the number of detections in each genus after BLAST-filtering. C) Number of identified proteins across methods (in 1–5, 6–8, and 9 in method 1). D) Number of identified proteins per subject and number of methods that identified the proteins.

The rate of bacterial protein misidentification across taxa has recently been evaluated in a benchmarking study by Rajczewski and colleagues.^52^ By systematically modifying the search database, they found that most bacterial protein misidentifications —particularly those based on single-peptide identifications— reflected reassignment to closely related species within the same genus when the correct species was absent from the database.^52^

Low abundance of bacterial proteins and inherent heterogeneity in clinical samples make it difficult to evaluate the reproducibility of the methods. The number of identified bacterial proteins per subject did not vary much (Figure 3D), but many bacterial proteins were identified with only one or two methods. Bacterial proteins composed 0.4% of the detected peptide intensities. There have not been many studies with a similar approach,^53,54^ so it is also difficult to cross-reference our findings. Kruh and colleagues^55^ conducted a study on the spleens of guinea pigs infected with mycobacteria and found that bacteria-to-host ratios of spectra (0.45%–0.81%) were in a similar range as ours. Altogether, our results show that bacterial proteins can be identified from the calcified coronary artery tissue.

There was no statistically significant difference in molecular weight, isoelectric point, or aliphatic index of the identified bacterial proteins between methods (). This shows that there is no systematic bias in the properties of the extracted bacterial proteins. Bacterial proteins consistently identified across the extraction methods were predominantly associated with core cellular and metabolic functions. Gene ontology enrichment pointed to primary metabolism and phosphorus-containing metabolic processes, reflecting the frequent detection of enzymes involved in nucleotide metabolism, redox homeostasis, and energy production. These protein classes are broadly conserved and typically highly abundant, making them more likely to be detected across different extraction workflows. Although these functions cannot be directly attributed to calcification processes, metabolic activities influencing phosphate turnover, redox balance, and cellular energy status have been connected with calcification-related processes in the literature^56^ as factors that may shape local microenvironments within mineralized tissues. Examples of proteins detected across multiple extraction approaches include fusobacterial thymidine kinase and streptococcal superoxide dismutase.

Methods 2b, 3, and 4 performed best in terms of the number of bacterial protein identifications. However, protein quantification failed for method 2b due to a low yield in a large portion of the samples. Furthermore, methods 1 and 2a yielded the lowest overall protein concentrations and produced the fewest bacterial protein identifications (Figure 3C). In contrast, despite large differences in total protein yield between methods 3 and 4, both yielded comparatively more bacterial protein identifications and the greatest number of proteins detected in more than 50% of the samples, indicating a more consistent recovery of bacterial proteins. High variability between the subjects may reflect individual differences in the stage of the atherosclerosis calcification process. As both were EDTA-based methods, these findings suggest that EDTA may be less suitable for recovering low-abundance bacterial proteins. Overall, method 3 proved to be the best extraction method in terms of the number of proteins identified, provided robust performance across biological samples as well as excellent high-throughput capacity for sample processing for a large number of samples.

### Fusobacterial Proteins Were Most Frequently Identified Bacterial Proteins

(Figure 4) Other frequently identified proteins included proteins from selected *Streptococcus* species, *Veillonella*, and *Prevotella*. Although Fusobacterium has been long considered to merely be a commensal bacteria, it has been linked with inflammation and immune evasion.^57^ Fusobacterium has an important role in the formation of oral biofilm formation. While early colonizers such as *Veillonella* and *Streptococcus* establish the initial microenvironment, Fusobacterium acts as a middle colonizer by providing a platform for the adherence of late colonizers *T. forsythia*, *A. actinomycetemcomitans*, *T. denticola*, and *P. gingivalis*.^58^

**Figure 4:**
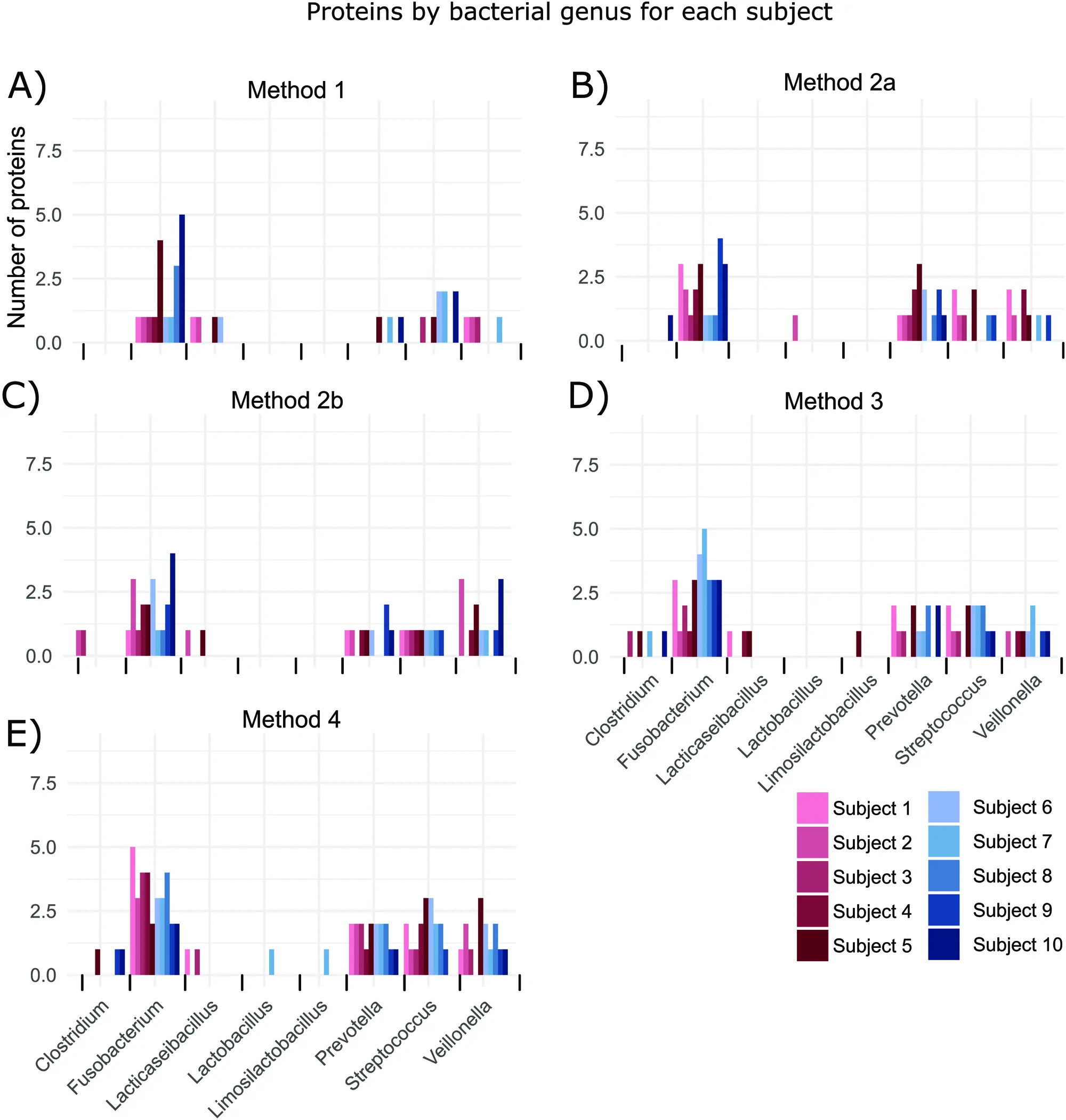
Subject-wise number of identified proteins in each genus for each method (A–E). Bacterial genera included in the search in the *x*-axis in alphabetical order.

## Concluding Remarks

Our analysis shows that proteins can be extracted from calcified coronary plaques with sufficient yield for MS analysis when appropriate extraction strategies are applied, enabling the detection of thousands of human proteins and two dozen bacterial proteins. The urea-based extraction method offered the most robust balance on several key metrics such as throughput capacity and number of identifications. Identification of bacterial proteins confirms that microbial signatures can be captured from calcified material, suggesting a role of bacteria in the inflammation of atherosclerotic plaque and supporting the future use of proteomics as a complementary approach for studying plaque composition. By enabling broad molecular profiling, global proteomics may help unravel the complex biological events that contribute to atherosclerotic plaque calcification.

## Supplementary Material





## Data Availability

Raw proteomics data and FASTA-libraries used in in-silico spectral library generation are available in PRIDE-repository with project accession PXD078118.

## Notes

Figure S1. Dot plots of differences in properties of identified proteins between methods. Figure S2. Additional upset plots for the detected proteins. List of used reagents and R packages. (PDF)
Musta et al. 2026 R scripts and original files. Reproducibility package containing all R scripts, source data, processed data, and intermediate result files used in the analyses and figure generation. Scripts are numbered according to execution order and include explicit input and output file definitions (ZIP)
