# Supplementary material for: Toward Optimized Proteomics Analysis to Detect Bacterial and Host Proteins in Calcified Coronary Artery Plaques
Source: J Proteome Res. 2026 Sep 14;25(10):5611–23. doi: 10.1021/acs.jproteome.6c00422 (PMC13640516; doi:10.1021/acs.jproteome.6c00422)
Supplement: Supplementary file 1 [file pr6c00422_si_001.pdf]

Supporting information

Towards optimized proteomics analysis to detect bacterial and host proteins in calcified coronary artery plaques

Kirsikka Musta[1]*, Markus J.T. Ojanen[1], Maria Stensland[2], Jonna Kankaanranta[1], Terho Lehtimäki[1,4,5], Pekka J. Karhunen[1,3,5], Tuula A. Nyman[2] and Vesa P. Hytönen[1,3]*

1: Faculty of Medicine and Health Technology, Tampere University, Tampere 33520, Finland

2: Department of Immunology, University of Oslo and Oslo University Hospital, Oslo 0372 Norway

3: Fimlab Laboratories, Tampere 33520, Finland

4: Department of Clinical Chemistry, Fimlab laboratories 33520, Tampere Finland

5: Finnish Cardiovascular Research Center - Tampere, Faculty of Medicine and Health Technology, Tampere University, Tampere 33520, Finland

*Correspondence to Kirsikka.musta@tuni.fi & vesa.hytonen@tuni.fi

Table of content

Figure S1 2

Figure S2 3

List of Reagents..3

List of all the used R-packages:..5

Used R-scripts and original files (zip)..6

Figure S1

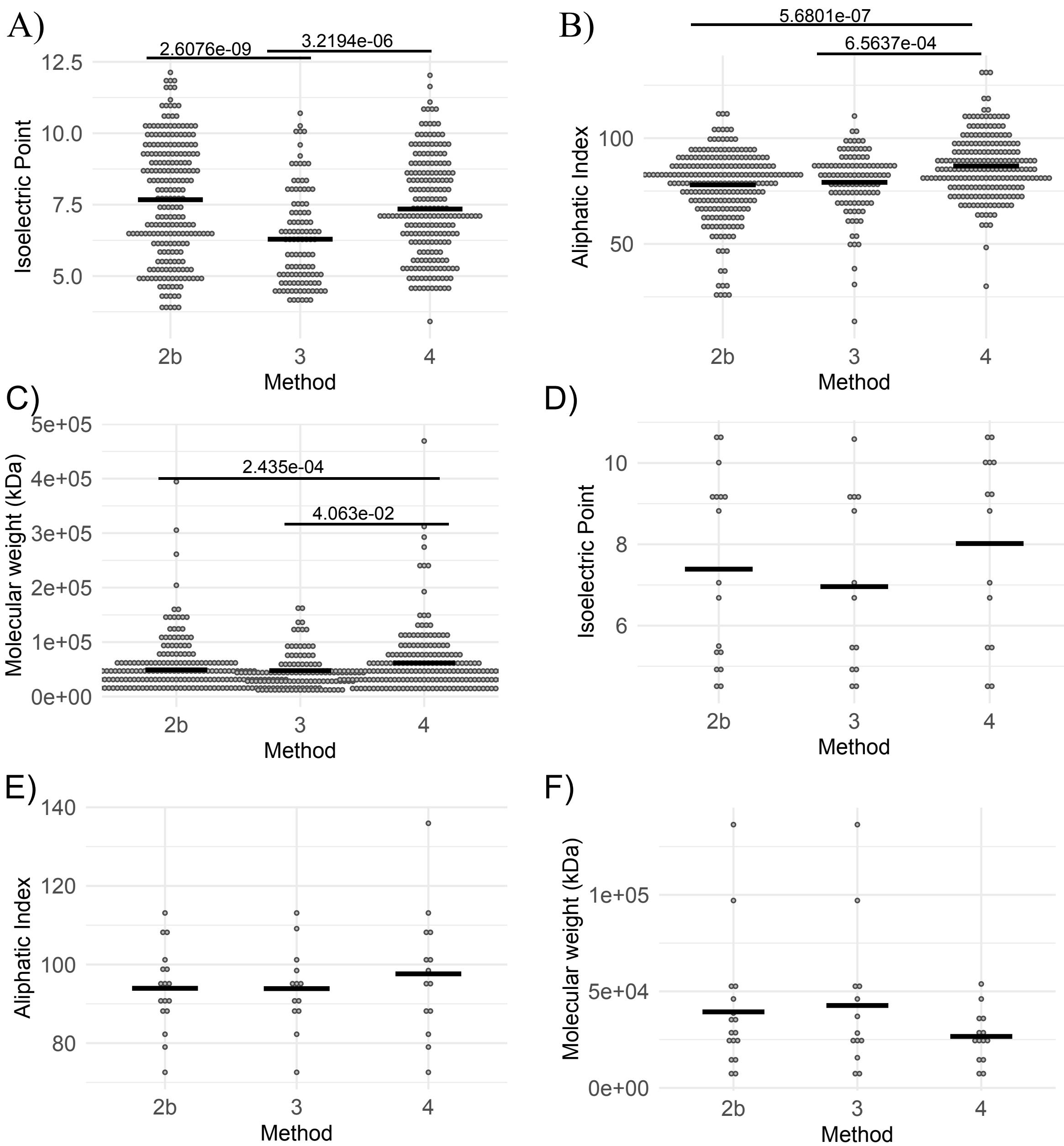

Figure S1. Dot plots of differences in properties of identified proteins between methods. A) Isoelectric point of proteins which were identified in all 10 samples by specific method B) Aliphatic index of proteins which were identified in all 10 samples by specific method C) Molecular weight (kDa) of proteins which were identified in all 10 samples by specific method D) Isoelectric point of bacterial proteins E) Aliphatic index of bacterial proteins F) Molecular weight (kDa) of bacterial proteins

Figure S2

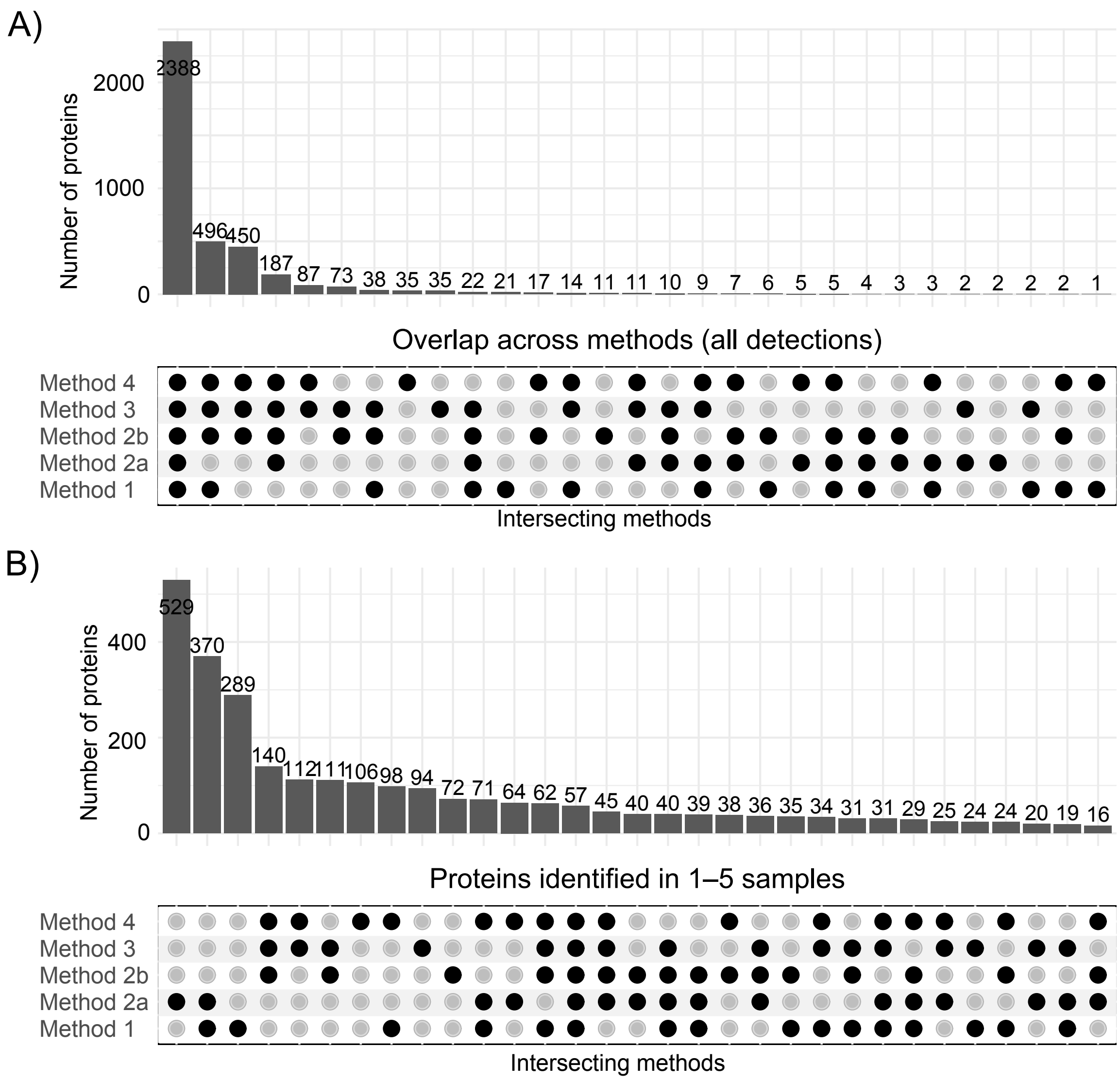

Figure S2. Upsetplots for the detected proteins. A) Overlap across methods for proteins detected in any number of samples B) Overlap across methods for proteins detected in 1-5 samples in each method

List of Reagents

Method 1 (Hendy et al., 2018)

- EDTA, 0.5 M – VWR Life Science, Cat# E522, United States
- B-PER (Bacterial Protein Extraction Reagent) – Thermo Fisher, Cat# 73243, United States
- SDS, 20% – CAT – Invitrogen by Thermo Fisher Scientific, Cat# AM9820, Lithuania

Method 2 (Palmer et al., 2021)

- EDTA, 0.5 M – VWR Life Science, Cat# E522, United States
- Guanidine Hydrochloride (GdnHCl), 6 M – Sigma-Aldrich, Cat# G3272
- Tris, 100 mM, pH 7.4 – Roche, Cat# 10812846001, Germany

Method 3 (Herrington et al., 2018)

- Urea, 8 M (freshly prepared) – Sigma-Aldrich, Cat# U5128, United States
- Thiourea, 2 M – Sigma-Aldrich, Cat# T8656, India
- CHAPS, 4% – Roche, Cat# 10810118001, Mannheim, Germany
- DTT, 1% – Roche, Cat# 10197777001, Mannheim, Germany

Method 4

- Zirconium beads – Macherey-Nagel, Cat# 740812.50
- Sodium deoxycholate, 5% – Sigma-Aldrich, Cat# D6750, New Zealand

PAC Purification & Protein Digestion

- AmineBeads – MagReSyn, Cat# MR-AMN005
- Acetonitrile, 70% – Fisher, Cat# A/0627/15
- Acetonitrile, 100% – Fisher, Cat# A/0627/15
- Ammonium bicarbonate, 50 mM – Merck, Cat# A6141, Germany
- IAA (Iodoacetamide), 550 mM – Merck, Cat# I6125, United States
- Trypsin – Thermo Scientific, Cat# 90057
- Formic acid, 0.1% – Fisher, Cat# A117

Protein Quantification & SDS-PAGE

- Pierce Microplate BCA Protein Assay Kit (reducing agent compatible) – Thermo Scientific, Cat# 23252, United States
- Spark® Multimode Microplate Reader – Tecan
- SDS-PAGE Gel – BIO-RAD, Cat# 4568126, United States
- Pierce Silver Staining Kit – Thermo Scientific, Cat# 24612

LC-MS/MS Analysis

- C18-tips – Thermo Scientific, Cat# 87784, United States
- MiVac concentrator – Genevac

List of all the used R-packages:

Auguie B (2017). _gridExtra: Miscellaneous Functions for "Grid" Graphics_. doi:10.32614/CRAN.package.gridExtra <https://doi.org/10.32614/CRAN.package.gridExtra>, R package version 2.3, <https://CRAN.R-project.org/package=gridExtra>.

Murrell P, Potter S (2025). _gridSVG: Export 'grid' Graphics as SVG_. doi:10.32614/CRAN.package.gridSVG <https://doi.org/10.32614/CRAN.package.gridSVG>, R package version 1.7-7, <https://CRAN.R-project.org/package=gridSVG>.

H. Wickham. ggplot2: Elegant Graphics for Data Analysis. Springer-Verlag New York, 2016. R package version 4.0.2

Wickham H, Bryan J (2025). _readxl: Read Excel Files_. doi:10.32614/CRAN.package.readxl <https://doi.org/10.32614/CRAN.package.readxl>, R package version 1.4.5, <https://CRAN.R-project.org/package=readxl>.

Wickham H, Averick M, Bryan J, Chang W, McGowan LD, François R, Grolemund G, Hayes A, Henry L, Hester J, Kuhn M, Pedersen TL, Miller E, Bache SM, Müller K, Ooms J, Robinson D, Seidel DP, Spinu V, Takahashi K, Vaughan D, Wilke C, Woo K, Yutani H (2019). “Welcome to the tidyverse.” _Journal of Open Source Software_, *4*(43), 1686. doi:10.21105/joss.01686 <https://doi.org/10.21105/joss.01686>.

Wickham H, François R, Henry L, Müller K, Vaughan D (2023). _dplyr: A Grammar of Data Manipulation_. doi:10.32614/CRAN.package.dplyr <https://doi.org/10.32614/CRAN.package.dplyr>, R package version 1.1.4, <https://CRAN.R-project.org/package=dplyr>.

Wickham H, Vaughan D, Girlich M (2024). _tidyr: Tidy Messy Data_. doi:10.32614/CRAN.package.tidyr <https://doi.org/10.32614/CRAN.package.tidyr>, R package version 1.3.2, <https://CRAN.R-project.org/package=tidyr>.

Wickham H (2025). _stringr: Simple, Consistent Wrappers for Common String Operations_. doi:10.32614/CRAN.package.stringr <https://doi.org/10.32614/CRAN.package.stringr>, R package version 1.6.0, <https://CRAN.R-project.org/package=stringr>.

Kassambara A (2025). _ggpubr: 'ggplot2' Based Publication Ready Plots_. doi:10.32614/CRAN.package.ggpubr <https://doi.org/10.32614/CRAN.package.ggpubr>, R package version 0.6.2, <https://CRAN.R-project.org/package=ggpubr>.

Carlson M (2025). _GO.db: A set of annotation maps describing the entire Gene Ontology_. R package version 3.21.0.

Müller K, Wickham H (2025). _tibble: Simple Data Frames_. doi:10.32614/CRAN.package.tibble <https://doi.org/10.32614/CRAN.package.tibble>, R package version 3.3.1, <https://CRAN.R-project.org/package=tibble>.

Osorio D, Rondón-Villarreal P, Torres R (2015). “Peptides: a package for data mining of antimicrobial peptides.” _The R Journal_, *7*(1), 4-14. doi:10.32614/RJ-2015-001 <https://doi.org/10.32614/RJ-2015-001>. R package version 2.4.6

Wickham H (2023). _httr: Tools for Working with URLs and HTTP_. doi:10.32614/CRAN.package.httr <https://doi.org/10.32614/CRAN.package.httr>, R package version 1.4.8, <https://CRAN.R-project.org/package=httr>.

Ooms J (2014). “The jsonlite Package: A Practical and Consistent Mapping Between JSON Data and R Objects.” _arXiv:1403.2805 [stat.CO]_. <https://arxiv.org/abs/1403.2805>.

Ooms J (2025). _writexl: Export Data Frames to Excel 'xlsx' Format_. doi:10.32614/CRAN.package.writexl <https://doi.org/10.32614/CRAN.package.writexl>, R package version 1.5.4, <https://CRAN.R-project.org/package=writexl>.

Kassambara A (2025). _rstatix: Pipe-Friendly Framework for Basic Statistical Tests_. doi:10.32614/CRAN.package.rstatix <https://doi.org/10.32614/CRAN.package.rstatix>, R package version 0.7.3, <https://CRAN.R-project.org/package=rstatix>.

Ogle DH, Doll JC, Wheeler AP, Dinno A (2025). _FSA: Simple Fisheries Stock Assessment Methods_. doi:10.32614/CRAN.package.FSA <https://doi.org/10.32614/CRAN.package.FSA>, R package version 0.10.1, <https://CRAN.R-project.org/package=FSA>.

Michał Krassowski. (2020). krassowski/complex-upset. Zenodo. http://doi.org/10.5281/zenodo.3700590

Alexander Lex, Nils Gehlenborg, Hendrik Strobelt, Romain Vuillemot, Hanspeter Pfister, UpSet: Visualization of Intersecting Sets, IEEE Transactions on Visualization and Computer Graphics (InfoVis'14), vol. 20, no. 12, pp. 1983–1992, 2014.

Used R-scripts and original files

Provided as a separate .zip-file (Musta et al. 2026 R scripts and original files)
